# Development of Nanopackaging for Storage and Transport of Loaded Lipid Nanoparticles

**DOI:** 10.1021/acs.nanolett.3c01271

**Published:** 2023-06-06

**Authors:** Apanpreet Kaur, Daniel Darvill, Shuning Xiang, Jerry Y. Y. Heng, Peter K. Petrov, Robert L. Z. Hoye, Rongjun Chen

**Affiliations:** † Department of Chemical Engineering, Imperial College London, South Kensington Campus, London SW7 2AZ, United Kingdom; ‡ Department of Materials, Imperial College London, South Kensington Campus, London SW7 2AZ, United Kingdom; § Inorganic Chemistry Laboratory, Department of Chemistry, University of Oxford, South Parks Road, Oxford OX1 3QR, United Kingdom

**Keywords:** nanohole storage arrays, drug delivery, quartz crystal microbalance with dissipation, functionalized lipid nanoparticles

## Abstract

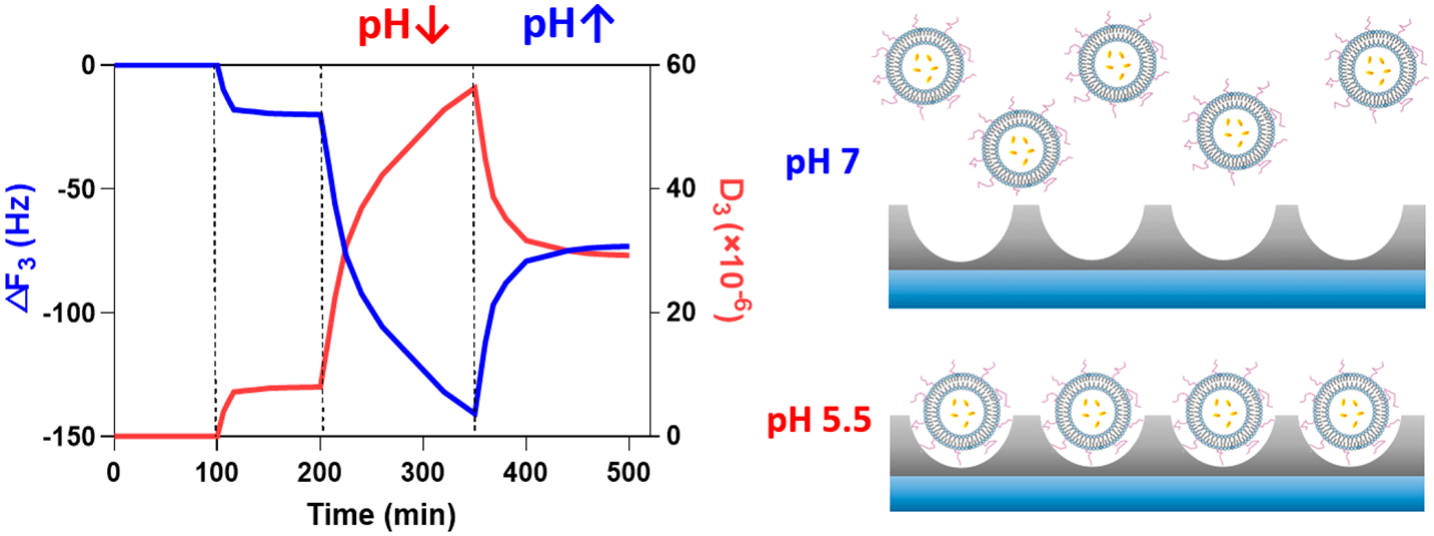

Easily deploying new vaccines globally to combat disease outbreaks has been highlighted as a major necessity by the World Health Organization. RNA-based vaccines using lipid nanoparticles (LNPs) as a drug delivery system were employed to great effect during the recent COVID-19 pandemic. However, LNPs are still unstable at room temperature and agglomerate over time during storage, rendering them ineffective for intracellular delivery. We demonstrate the suitability of nanohole arrays (nanopackaging) as patterned surfaces to separate and store functionalized LNPs (fLNPs) in individual recesses, which can be expanded to other therapeutics. Encapsulating calcein as a model drug, we show through confocal microscopy the effective loading of fLNPs into our nanopackaging for both wet and dry systems. We prove quantifiably pH-mediated capture and subsequent unloading of over 30% of the fLNPs using QCM-D on alumina surfaces altering the pH from 5.5 to 7, displaying controllable storage at the nanoscale.

The recent COVID-19 pandemic has shown the worldwide need for a rapid response to protect against severe disease outbreaks. Messenger ribonucleic acid (mRNA) vaccines have been demonstrated as highly effective at treating COVID-19 and are being developed to combat other infectious diseases, such as the Ebola virus disease, influenza, Zika virus, and human immunodeficiency virus (HIV).^1^ However, many physical and economical challenges, such as the stability and shelf life of these new vaccines, remain as critical obstacles to the rapid and widespread use of mRNA-based vaccines. Current mRNA vaccines require refrigeration after defrosting and need to be used within 6 to 24 h of being brought to room temperature. The issue of these storage conditions is that they represent a large cost in cold chains, which are supply routes for the transport and storage of these vaccines to areas where they are required. Compared to DNA, mRNA is weaker in structure and more prone to thermal degradation and breakdown due to the highly reactive hydroxyl groups on the backbone of the structure’s backbone. Such vaccines can require storage between −90 and −15 °C, lasting 9 months, followed by refrigeration at 2–8 °C, lasting up to one month, without the possibility of refreezing.^2^ Practicality and high cost can make such vaccines difficult to distribute to hard-to-reach areas and countries where these cold chains are not in place, particularly developing nations.^3,4^

Lipid nanoparticles (LNPs) are a novel drug delivery system of nanoparticle shells composed of lipids to encapsulate unstable vaccines for delivery into humans, first evidenced with the Moderna and Pfizer-BioNTech COVID-19 vaccinations.^5−8^ Although conventional LNPs can encapsulate various payloads including RNA-based vaccines, improving their stability, they are also prone to the same degradation mechanisms due to changes in pH, temperature, and enzymatic interactions with each other and their excipients, degrading the mRNA. Besides degradation, LNPs in solution may also agglomerate, reaching a critical size where they may no longer be internalized into the cell and can suffer from having poor solubility, high immunogenicity, and rapid clearance, limiting clinical applications. Different approaches to overcome mRNA degradation may include optimizing the mRNA designs, additional functionalization of the LNPs, or freeze-drying to remove water.^9−11^ In this paper, we investigate the combination of further polymer functionalized LNPs (fLNPs) with the storage of individual LNPs in separate recesses of a nanohole array, which we propose as a novel approach to mitigate agglomeration.

Nanohole arrays are an already well-established and well-explored structure in the fields of light–matter interaction due to their extraordinary optical transmission and plasmonic properties for sensing and light harvesting.^12−16^ To date, however, there is no literature relating to the use of these nanoholes as a nanopackaging to separate, stabilize, and store nanoformulations. This approach will allow us to overcome issues of agglomeration for fLNPs in dry systems, such as freeze-drying, or other approaches to dry and store LNPs long term at room temperature,^17^ or store in solution (i.e., wet systems), for example after defrosting. Monodisperse LNPs will lead to better predictive payload release kinetics and favorable *in vivo* distribution by mechanisms, such as escape from the endothelium through the nanomaterials induced endothelial leakiness (NanoEL) effect, which are size dependent.^18^ We hypothesize that this nanopackaging will also improve LNP shelf life as the nanohole side walls containing the LNPs will protect against degradation from contact with other constituents and LNPs in solution. Similar works for fabricating nanohole arrays on the micrometer scale have been used to separate cells into arrays for analysis, where loading and unloading of these cells are achieved mechanically, electrostatically, or thermophoretically.^19,20^ We propose and investigate whether the loading and unloading of fLNPs from nanohole arrays can be controlled by altering the pH of the solution, changing the isoelectric point of the surface, and manipulating the electrostatic interactions between the surface and the LNPs.

The LNPs used in this study are surface-modified multifunctional LNPs using viral-peptide-mimicking pH-responsive anionic biopolymers, which provide a more controlled delivery. Novel polymers have been developed by Chen et al.^21,22^ and have successfully shown endosomolytic activity and cytoplasmic delivery of drug payloads.^23^ One of the superior polymers, PP75, has been used on the surface of the LNPs made of zwitterionic phospholipid, 1,2-dioleoyl-*sn*-glycero-3-phosphoethanolamine (DOPE), and cholesterol to form functionalized LNPs (fLNPs). PP75 is based on side-chain modification of a metabolite-derived poly(carboxylic acid) polymer backbone, poly(l-lysine isophthalamide) (PLP), with l-phenylalanine at 75% stoichiometric degree of substitution. l-Phenylalanine is a hydrophobic amino acid found in fusogenic viral peptides of the influenza virus. These pH-responsive pseudopeptidic polymers are of interest, as they can mimic the anionic peptides on the surface of viruses that play a role in membrane destabilization, enhancing intracellular delivery.

The physiochemical characterization of these fLNPs encapsulating calcein as the model drug for loading in our nanopackaging is shown in Figure 1. The synthesis process and additional details of the characterization can be found in the Supporting Information. The effect of polymer concentration on the hydrodynamic diameter of the fLNPs is given in Figure 1A. The average particle size was found to be 201 ± 9 nm for varying concentrations of PP75 polymer coating with a narrow size distribution polydispersity index (PDI) < 0.4, suggesting the fLNPs were monodisperse. There is no significant size difference between PP75-coated and uncoated LNPs.^23^ This is ideal for intracellular delivery as particles with hydrodynamic diameters of ∼200 nm or below have been shown to effectively enter cells via clathrin-coated pits in the cell membrane.^24^ The surface charge of the particles, as shown by the zeta potential data in Figure 1B, varies from −1.7 ± 0.3 mV for uncoated LNPs to −20.8 ± 1.3 mV for fLNPs with 20 μM PP75, owing to the surface coating with PP75 containing one carboxyl group pendant to each polymer unit. The negative charge is beneficial for vaccine applications, as drainage into lymph nodes is enhanced, triggering higher levels of antibody production compared to cationic particles.^25^

**Figure 1:**
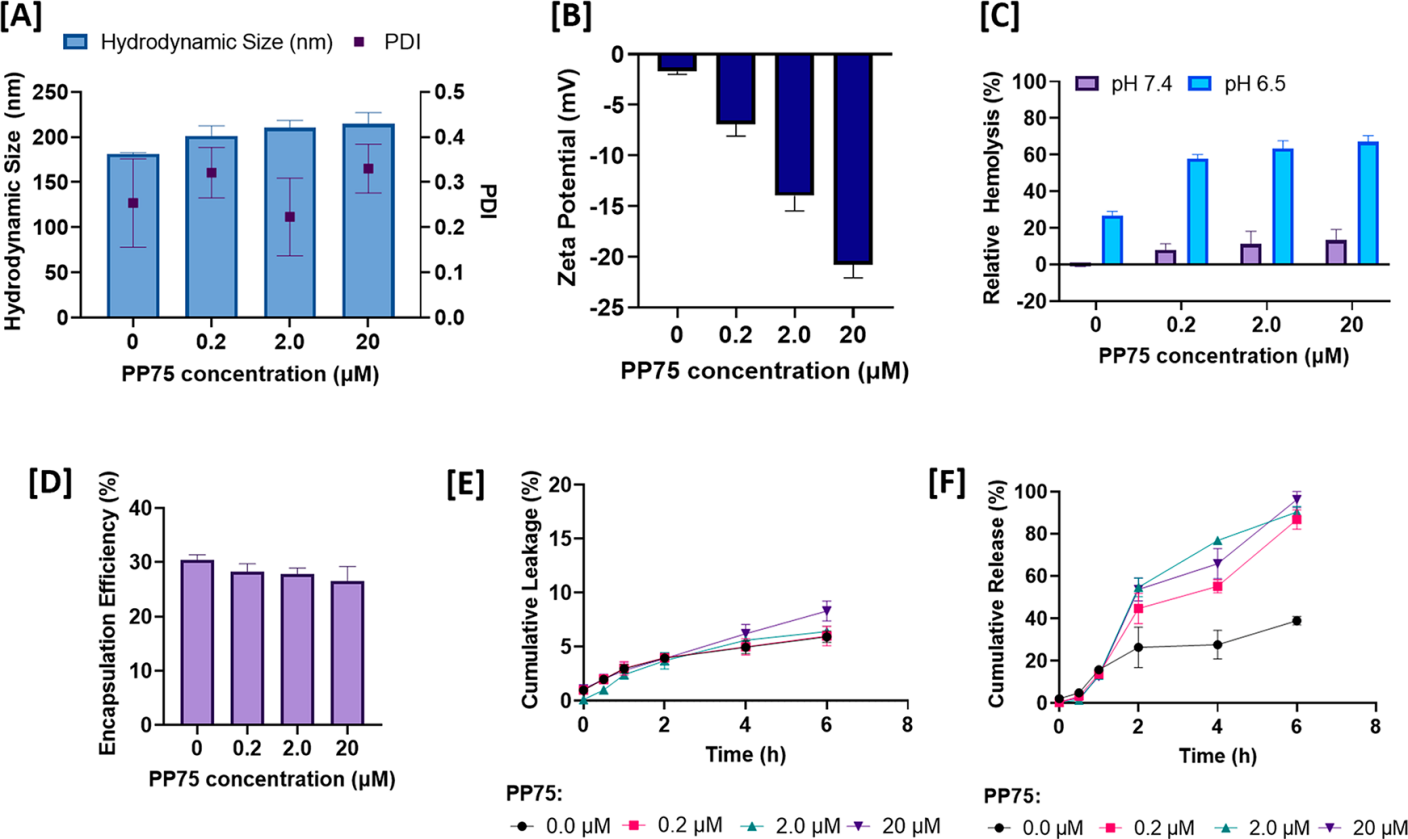
Characterization of fLNPs with varying concentrations of PP75 polymer surface coating: [A] mean hydrodynamic size and polydispersity index (PDI), [B] zeta potential measurements, and [C] relative hemolysis of RBCs incubated with fLNPs with PP75 at various concentrations for 1 h, at pH 7.4 and 6.5. [D] Effect of PP75 polymer concentration on the encapsulation efficiency of calcein. [E] Cumulative leakage of calcein from fLNPs at varying concentrations of PP75 polymer coating, at pH 7.4, over 6 h at room temperature. [F] Calcein release from fLNPs at varying concentrations of PP75 polymer coating, at pH 6.5, over 6 h at room temperature. Mean ± SD (*n* = 3).

Cell membrane destabilization using a hemolysis assay was performed on sheep red blood cells (RBCs) as a model of endosomes to examine the ability of the PP75-coated fLNPs to trigger endosomal escape.^26^Figure 1C shows that as the pH decreased, from physiological pH (pH 7.4) to early endosomal pH (pH 6.5), the relative hemolysis of the fLNPs increased considerably. An effective delivery system should have low hemolysis at physiological pH and the ability to destabilize the endosomal membrane when acidification in the endosomal compartments takes place.^23^ The pH-responsive hemolystic profile of the PP75-coated fLNPs was comparable with PP75 alone (Figure S1), as the polymer is pH-responsive and changes in structure, from coiled to globular when pH is reduced below its p*K*_a_, destabilizing the membrane at endosomal pH.^21^ Moreover, the relative hemolysis of the fLNPs increased to 67.1 ± 3.3% as the polymer concentration increased at endosomal pH 6.5. This suggests that PP75 triggers endosomolytic activity at mildly acidic pH, making the fLNPs suitable candidates for efficient intracellular delivery of various payloads.

We then tested the encapsulation efficiency of fLNPs containing calcein as our model drug, varying the PP75 concentration, as shown in Figure 1D. The encapsulation efficiency remained consistent, averaging at 28.3 ± 1.5%, as expected for passively loaded payloads. The consistency across different polymer concentrations is due to the presence of 40 mol % cholesterol in the fLNPs, which condenses lipid packing in the system, making the membrane more rigid.^27,28^ This also reduces the permeability of the system, allowing for more stable encapsulation efficiencies and reduced leakage at pH 7.4, despite surface modification.^29^ The favorable minimal leakage of calcein, 6.7 ± 0.7%, from the fLNPs for different PP75 concentrations over 6 h at pH 7.4 is shown in Figure 1E. The fLNPs display no initial signs of aggregation, sedimentation, or instability at physiological pH.

Lastly, pH-triggered payload release from fLNPs with the varying PP75 concentration at endosomal pH is shown in Figure 1F. At pH 6.5 characteristic of early endosomes, the calcein release after 6 h was 38.9 ± 2.0% for the uncoated LNPs and 86.7 ± 4.6%, 90.3 ± 2.6%, and 96.2 ± 3.8% for the fLNPs coated with 0.2, 2.0, and 20 μM PP75, respectively. This pH-responsive payload release profile of the PP75-coated fLNPs is favorable for intracellular delivery. The increased driving force for release of calcein increased at pH 6.5 because of the enhanced interaction between PP75 and the lipid membrane upon acidification. The conformation of PP75 depends on the balance between electrostatic repulsion and hydrophobic association.^22,23,30^ At pH 7.4, the electrostatic repulsion between weakly charged carboxyl groups is dominant; this results in PP75 having an expanded coil structure with low membrane activity, which is consistent with the minimal calcein leakage at pH 7.4, as shown in Figure 1E. At pH 6.5, carboxyl groups are protonated, leading to the polymer forming a hydrophobic, globular structure with significantly enhanced membrane activity.^23,31^ This leads to PP75 interacting deeper into the lipid bilayer, causing the formation of pores, which causes calcein release.^21,32^

From these results, it was decided that the fLNPs with a PP75 polymer coating of 20 μM would be ideal to use in this study. This enables the highest endosomolytic activity and pH-triggered payload release, while maintaining a stable and monodisperse structure with minimal leakage over 6 h.

Second, we show the fabrication of our nanohole array for use as nanopackaging. Polystyrene nanospheres, diameter 488 ± 5 nm, were first deposited as a hexagonal close-packed (hcp) monolayer by a well-known colloidal lithography “fishing” process on silicon substrates to create a colloidal mask^33−36^ (see Figure 2A). We note the presence of grain boundaries where there is nonperfect long-range order, but there are no large defects, and it is shown below that these grain boundaries do not affect the loading of fLNPs into the nanohole array. Although the density of nanoholes may be lower than that of a perfectly ordered colloidal mask during deposition, the technique used is both cost-effective and facile. There are no requirements for any advanced processing equipment to produce colloidal masks on any desired substrate materials, and they have the potential to be scaled up.

**Figure 2:**
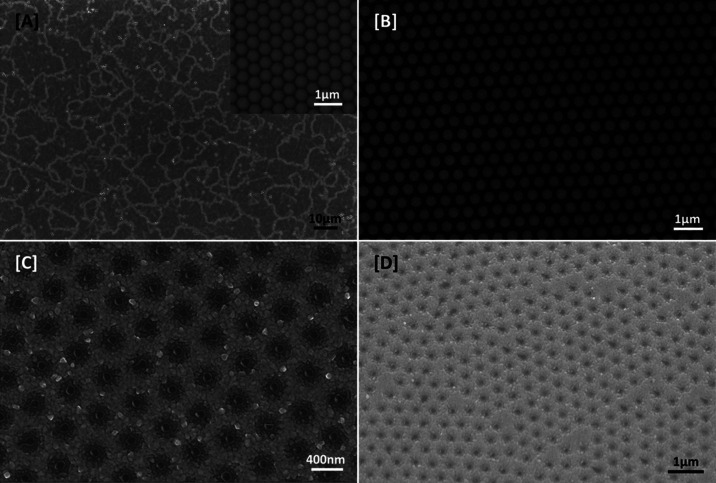
Fabrication of nanohole arrays by colloidal lithography. SEM images of a polystyrene (PS) mask on glass, [A] before and [B] after oxygen plasma treatment; scale bars 10 and 1 μm, respectively. The inset of [A] shows PS packing before treatment; scale bar is 1 μm. [C] Top-down and [D] 30° tilt SEM image of nanohole array after the deposition of the titanium adhesion layer and aluminum thin film and after PS mask removal; scale bars 400 nm and 1 μm, respectively.

Before deposition, the colloidal mask was first treated in oxygen plasma to homogeneously etch and shrink the PS nanospheres to spatially separate them. The average diameter of the etched PS spheres was found to be approximately 388 ± 5 nm (see Figure 2B). Afterward, DC sputtering of an 8 nm thick titanium adhesion layer, followed by deposition of a 142 nm thick aluminum layer was performed. After deposition, the colloidal mask was removed by mechanical exfoliation to produce the nanohole array seen in Figure 2C,D. The nanohole array recesses follow a concave shape with an outer edge diameter of 388 nm curving down to a flat inner bowl diameter of 100 nm. Details of the fabrication process may be found in the Supporting Information.

We then demonstrate the filling of the nanohole array with calcein-loaded fLNPs for both a wet and dry system, as shown in Figure 3. In Figure 3A, a confocal microscopy image of the loading of the fLNPs into the nanopackaging as a wet system is shown. The fLNPs were drop-cast in excess of those required to fill every nanohole on the substrate surface and allowed to settle for 1 h, followed by a lateral washing step to remove excess fLNPs. From Figure 3A we can see the successful loading of the fLNPs and that they are separated into individual nanoholes on the surface. The circular patterns on the image relate to the diffraction of oil in contact with the coverslip; details of the loading and imaging conditions can be found in the Supporting Information. In Figure 3B, a dry system where fLNPs were loaded by spin coating into the nanopackaging and spun until dry is shown. As with the wet system, this shows successful separation and loading of the fLNPs into individual nanoholes, while excess LNPs are removed from the surface at high spin speeds. We note the presence of regions with no fLNPs loaded, which correspond to the grain boundaries of the colloidal mask, discussed for Figure 2A. Where no nanohole arrays are patterned, i.e. a flat surface, there is no successful storage of fLNPs. The same loadings were performed for flat alumina surfaces under the same conditions, and there was an absence of fLNPs for both the wet and dry systems, as shown for an example of the dry system in Figure S2A. To further demonstrate the loading of the fLNPs, SEM images of fLNPs loaded into the nanopackaging for a magnified and lower magnification image are shown in Figures 3C and 3D, respectively. The samples were first coated with 10 nm of gold for imaging purposes, and some deformation of the fLNPs is noted due to the samples being under vacuum before deposition began. Finally, to prove that it is also possible to remove the fLNPs from loaded nanopackaging, we washed the sample multiple times with Dulbecco’s phosphate-buffered saline (DPBS) solution after confocal imaging and found that there were no longer any fLNPs present, as shown in Figure S2B. Optical images corresponding to the confocal images taken may also be found in Figure S2.

**Figure 3:**
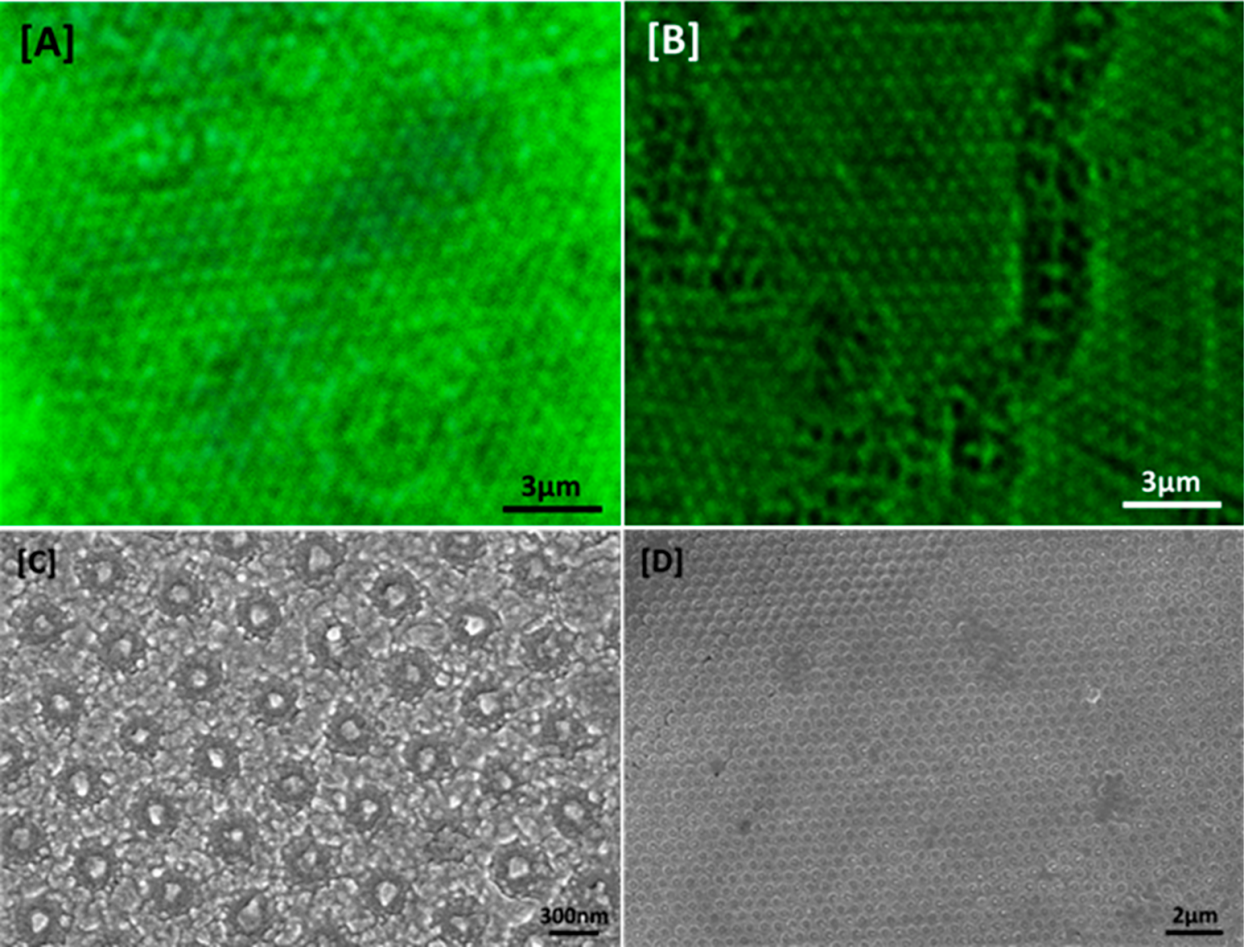
Confocal microscopy images of PP75-coated, calcein-encapsulated fLNPs loaded into nanohole arrays for both a [A] wet and [B] dry system; scale bars 3 μm. [C] SEM image of fLNPs loaded into nanopackaging coated with a gold film and [D] a lower magnification image of loaded fLNPs for the dry system; scale bars of 300 nm and 2 μm, respectively.

Having confirmed that we can load and unload fLNPs from nanopackaging, it is important to show that we can achieve this process in a quantifiable and controlled way. We show pH-mediated control of this process in solution to demonstrate the loading and unloading conditions of the fLNPs for different material surfaces. In aqueous solution, the effective surface charge state of a material is dependent on the interactions of this surface with the ions in the solution. In water when the concentrations of H^+^ and OH^–^ ions that adsorb onto the materials’ surface create a zero point charge, this is termed as the isoelectric point for the material.^37^ Oxide materials were considered for the nanohole array as many have isoelectric points in the range of pHs we are interested in for loading and unloading, while keeping the fLNPs stable. When, for example, an oxide surface is immersed in aqueous solutions with a pH higher than the isoelectric point of the oxide, the surface hydroxyl groups dissociate and thus the surface becomes negatively charged.^38^ By then choosing a material that exhibits this change in surface charge in our pH range of interest, we can load and unload the negatively charged fLNPs. Of these materials, aluminum oxide was chosen as the deposition material as it is well-studied, biocompatible, and has been used for other drug delivery systems which are pH sensitive.^39−41^

Changing the pH, we explored whether the electrostatic interactions play a role in the loading and unloading processes of the fLNPs. QCM-D was used to compare the loading conditions of fLNPs without drug encapsulation for silica and alumina surfaces, while altering the pH. Using as-purchased quartz and alumina coated sensors, we monitored changes in frequency (Δ*F*_3_) and dissipation (Δ*D*_3_) in the third overtone, 3 times the frequency of the crystal. Further details may be found in the Supporting Information. A lower limit pH of 5.5 was selected, as PP75 will change its conformation from a hydrophilic coil structure to hydrophobic globular structure at pH ∼6.1, while remaining higher than the precipitation point of the polymer.^22,42^ pH 6.0 was chosen for the loading onto alumina due to its higher isoelectric point (6.4–6.7).^43^ The frequency changes achieved by the QCM-D are shown in Figure 4. At neutral pH, the fLNP loading process reached equilibrium in around 1 h. As pH decreased, the frequency change increased dramatically, and the loading did not stop after 3 h. The adsorption of fLNPs at pH 7.0 and 5.5 in 2 h on the two different surfaces was compared and showed a lower mass loading at a higher pH (see Figure 4A). This is mostly due to the aggregation of the fLNPs in the acidic environment. At neutral pH, the carboxylate groups contained within PP75 will be deprotonated, and the repulsive force among the anions leads to its extended polymer conformation. When decreasing the pH from 7.0, the carboxylate groups of PP75 polymer will be protonated, leading to a globular conformation. The fLNPs coated with PP75 will then become connected with each other; the process is reversible.^23^ This property of PP75 helps to load fLNPs in an acidic environment. A potential challenge could be release of small-molecule payloads at mildly acidic pH, as shown in Figure 1C. It should not be a problem though for a macromolecular payload like RNA that is encapsulated into the LNP core via electrostatic interactions.

**Figure 4:**
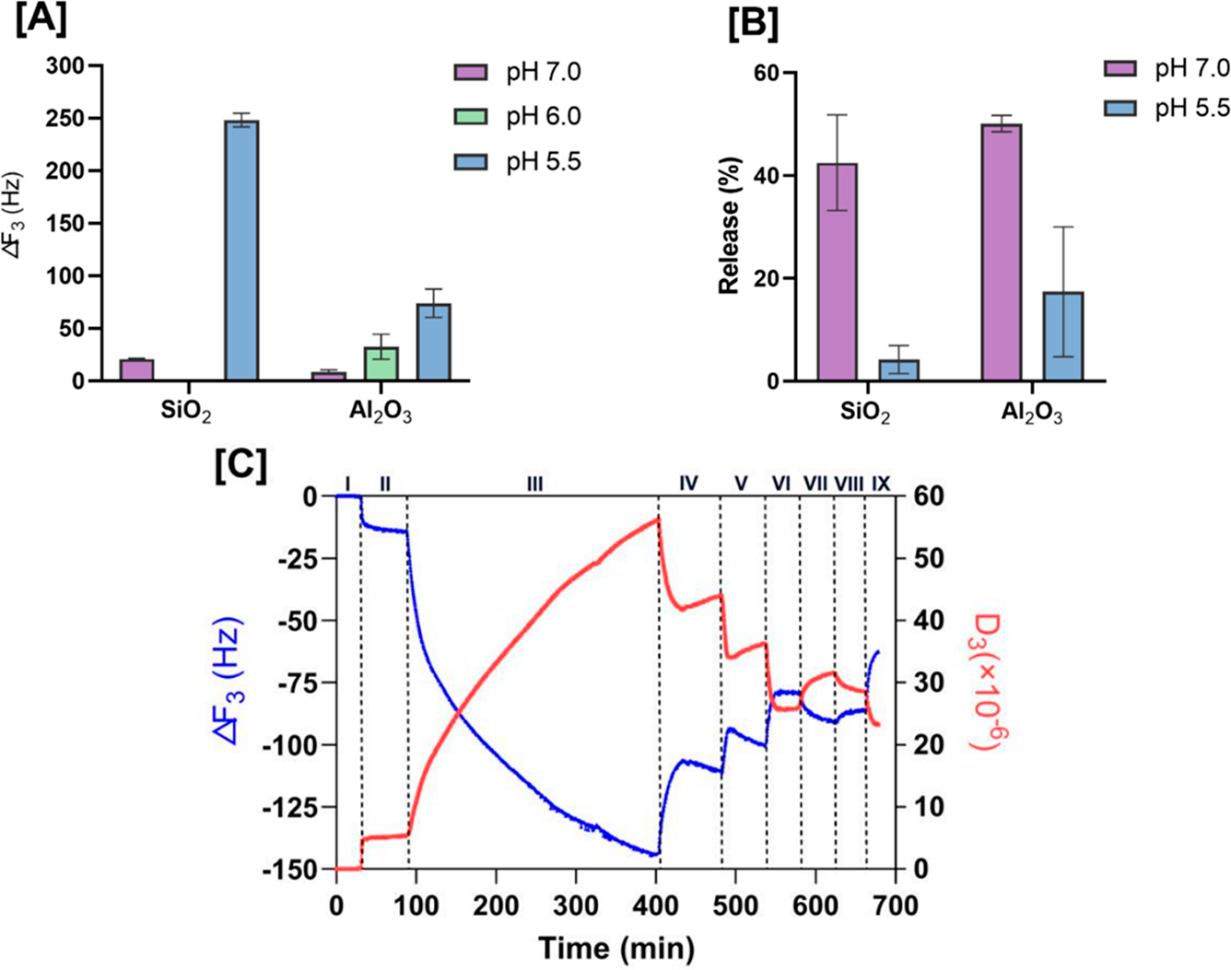
[A] Frequency changes of the third overtone of the quartz crystal microbalance (Δ*F*_3_) with dissipation monitoring (QCM-D) from the loading of 0.8 × 10^9^ particles/mL 200 nm fLNPs with 20 μM PP75 on two different flat surfaces (silica and alumina) at pH 7, 6, and 5.5 over the course of 2 h. [B] Percentages of the desorption of fLNPs by flowing DPBS buffer at the same pH as adsorption on two different flat surfaces. [C] Frequency (Δ*F*_3_, blue) and dissipation (Δ*D*_3_, red) changes of the third overtone during fLNPs loading and unloading on the alumina surface in (I) deionized water (dH_2_O), (II) DPBS at pH 5.5, (III) 0.8 × 10^–9^ particles/mL 200 nm DOPE + 20 μM PP75 at pH 5.5, (IV) DPBS at pH 5.5, (V) DPBS at pH 6, (VI) DPBS at pH 7.0, (VII) DPBS at pH 5.5, (VIII) DPBS at pH 7.0, and (IX) dH_2_O.

When directly comparing the two QCM materials, the adsorption of fLNPs on hydrophilic silica and hydrophobic alumina in a flowing system is more than that on alumina. This difference is more significant at pH 5.5 (see Figure 4A). The alumina surface is positively charged at pH 5.5, as it is lower than its isoelectric point, and we expect stronger interaction with the negatively charged fLNPs than that with the silica surface. The results deviate from expectations, which indicates that electrostatics are not the main interactions between the fLNPs and the surface. The salts (Mg^2+^, Ca^2+^, Na^+^, and K^+^) in the buffer solution form ion bridges between both negatively charged fLNPs and the surface.^43^ The formation of hydrophobic microdomains for PP75 at low pH may therefore play a role.

After adsorption of fLNPs, DPBS-only solution at the same pH flowed through the QCM-D module. The results indicated that the fLNPs can be easily removed from the surfaces, and a higher percentage of fLNPs can be unloaded at neutral pH than at pH 5.5, as seen in Figure 4B. In Figure 4C a complete time trace of the loading and unloading of fLNPs from an alumina surface is given, allowing time for the adsorption of LNPs and stabilization of the QCM, followed by the subsequent unloading of fLNPs at a higher pH. By stepping the changes in pH of the DPBS solution with time, we can see the step unloading of fLNPs from the QCM surface. From this we can infer that the adsorption of fLNPs at lower pH can be released at a higher pH.

In conclusion, we have successfully devised reproducible approaches to loading and unloading individual fLNPs into each nanohole in an array. We have shown effective loading of our polymer-functionalized PP75-coated, calcein-encapsulated fLNPs into individual recesses of nanohole arrays for both a wet and dry system and readily unloading by simple washing, as visualized by confocal microscopy. In addition, in choosing alumina as our desired nanohole array material, we have shown quantifiably by QCM-D that by altering the pH from 5.5 to 7 it is possible to control the reversible adsorption and desorption of fLNPs from the surface. This study shows an alternative approach that may be adopted as a method to improve storage at the nanoscale for other therapeutics and disciplines.

## Supporting Information Available

Materials and methods; fabrication of nanohole arrays, synthesis of functionalized LNPs, characterization of functionalized LNPs, loading of LNPs into nanohole array, QCM loading and optical characterization; supporting data; PP75 only hemolysis assay, confocal images of flat alumina surface and nanopackaging after washing, and supporting optical microscopy images of confocal imaging (PDF)

## Abbreviations

LNP: lipid nanoparticles
mRNA: messenger ribonucleic acid
PS: polystyrene
hcp: hexagonally close-packed
SEM: scanning electron microscopy
fLNP: polymer-functionalized lipid nanoparticles
DOPE: dioleoylphosphatidylethanolamine
PP75: l-phenylalanine (75 mol %)
PDI: polydispersity index
RBC: red blood cells
QCM-D: quartz crystal microbalance with dissipation monitoring
DPBS: Dulbecco’s phosphate-buffered saline
