# Supplementary material for: Development of Nanopackaging for Storage and Transport of Loaded Lipid Nanoparticles
Source: Nano Lett. 2023 Jun 6;23(14):6760–7. doi: 10.1021/acs.nanolett.3c01271 (PMC10375581; doi:10.1021/acs.nanolett.3c01271)
Supplement: Supplementary file 1 — nl3c01271_si_001.pdf [file nl3c01271_si_001.pdf]

SUPPORTING INFORMATION

# *Development of Nanopackaging for Storage and Transport of Loaded Lipid Nanoparticles*

*Apanpreet Kaur[1] ‡, Daniel Darvill[2,3] ‡, Shuning Xiang[1] ‡, Jerry Y. Y. Heng[1*], Peter K. Petrov[2*], Robert L. Z. Hoye[3,2*], Rongjun Chen[1] **

[1]Department of Chemical Engineering, Imperial College London, South Kensington Campus, London, SW7 2AZ, United Kingdom

[2]Department of Materials, Imperial College London, South Kensington Campus, London, SW7 2AZ, United Kingdom

[3]Inorganic Chemistry Laboratory, Department of Chemistry, University of Oxford, South Parks Road, Oxford, OX1 3QR, United Kingdom

*****E-mail: jerry.heng@imperial.ac.uk (J. Y. Y. H.), p.petrov@imperial.ac.uk (P. P.), robert.hoye@chem.ox.ac.uk (R. L. Z. H.), rongjun.chen@imperial.ac.uk (R. C.)

## Materials and Methods

### Fabrication of Nanohole Arrays

An air-water interface colloidal lithography ‘fishing’ technique was used to create the hcp PS monolayers. In summary, PS spheres with diameters of 488 nm monodispersed in 10 wt % aqueous solution were purchased from Bangs Laboratory. These were diluted in a 1:1 ratio with ethanol and shaken to create a disperse solution of PS spheres. A Si wafer was UV ozone treated (Ossila UV ozone machine) for 15 minutes to ensure a completely hydrophilic surface charge. This wafer

was then suspended at an angle of 80o with one edge of the wafer submersed into a glass bath filled with deionized water. The PS solution was then pipetted in lines onto the Si wafer so as to cover the wafer, whilst excess polystyrene flowed into the waste bath, and was allowed to dry. This created a monolayer of PS spheres on the wafer, which was vertically and controllably submersed into a second plastic water bath filled with DI water. This transferred the PS monolayer as a dense hcp monolayer onto the water surface at the air-water interface. The pH of the second bath was altered to pH 9 by addition of 1M sodium hydroxide solution to encourage compression of the PS mask during transfer due to creating a higher interfacial surface tension. This transfer process was repeated until the second water bath surface was covered in a PS monolayer. Desired substrates were then submersed beneath the water surface and used to 'fish' PS from the surface and dried ambiently at a 45$^{o}$ angle. If insufficient PS was used to fill the second bath surface, or after transfer of some substrates, a few μL of sodium dodecyl sulfate can be injected into the water-air interface to compress the remaining PS, keeping the surface tension sufficient to prevent loss of the PS hcp arrangement.

The hcp PS monolayer covered substrates were then treated with reactive ion etching to reduce the size of the PS spheres and separate them. The etching power, working distance and oxygen flow rate were fixed at 100 W, 5 cm and 20 sccm respectively. Following preparation of the colloidal mask samples were loaded into a Hex DC sputtering system and an 8 nm adhesion layer of titanium was deposited following a deposition of 142 nm of aluminium to create the nanohole arrays as measured by QCM. Finally, tape stripping was used to remove the PS mask from the surface.

Synthesis of Functionalized LNPs

## Materials

1,2-Dioleoyl-sn-glycero-3-phosphoethanolamine (DOPE) lipids, cholesterol, iso-phthaloyl chloride, fluorescein isothiocyanate and calcein were purchased from Sigma-Aldrich (Dorset, UK). N,N-dimethylformamide (DMF), triethylamine, sodium chloride and 4-dimethylaminopyridine (DMAP) were purchased from Fisher Scientific (Loughborough, UK). Anhydrous ethanol, acetone, hydrochloric acid, potassium carbonate, sodium hydroxide, diethyl ether and chloroform were obtained from VWR (Lutterworth, UK). L-lysine methyl ester dihydrochloride, L-phenylalanine methyl ester hydrochloride and N,N'-dicyclohexylcarbodiimide (DCC) were purchased from Alfa Aesar (Heysham, UK). Dulbecco's phosphate-buffered saline (DPBS) were purchased from Sigma-Aldrich (Dorset, UK). Defibrinated sheep red blood cells (RBCs) were purchased from TCS Biosciences Ltd (Buckingham, UK). Finally, the polymers, Poly(L-lysine isophthalamide) (PLP) and PP75, a PLP side chain grafted on L-phenylalanine at a stoichiometric ratio of 75% were synthesized in-house using protocols developed by Eccleston et al. (2000)[1] and Chen et al. (2009)[2], respectively.

## Preparation of Lipid Solution

A lipid film was prepared according to the method used by Guo et al., (2015).[3] Specifically, DOPE and 40 mol% cholesterol were dissolved in chloroform. 1% (v/v) ethanol was added to DOPE. The solvent was removed by rotary evaporation over a 3-hour period. This formed a thin lipid film inside a round bottom flask. The film was hydrated in a water bath at 30 C for 1 h.

## Liposome Synthesis and Calcein Encapsulation

Sonication (Sonicator, 120 watt, Fisher Scientific) was used to reduce the hydrodynamic size of the LNPs for 10 minutes at 50% amplitude, with 1 second on and 1 second off pulsation. At this point, Calcein was added for in-situ encapsulation. After, the loaded LNPs were filtered through a 0.22 μm filter and stored at 4 ˚C.

**Formation of Functionalized LNPs**

To determine polymer coating efficiency on the surface of the LNPs, FITC-PP75 was used to coat LNPs at known concentrations. 10 mg $mL^{-1}$ stock solution was made using DPBS, at pH 7.4, which was diluted to desired concentrations. This was mixed with the liposome solutions and left to adsorb overnight. The excess PP75 was removed using dialysis devices (Float-A-Lyzer®, MWCO 300 kDa, Spectrumlabs, USA). The fluorescence was measured using a Spectrofluorometer (GloMax®-Multi Detection System, Promega, USA), with excitation wavelength at 490 nm and emission wavelength 510 to 570 nm. A calibration line was plotted using known concentrations of FITC-PP75 at pH 7.4 to convert fluorescence readings to concentration.

Characterization of Functionalized LNPs

**Dynamic Light Scattering (DLS)**

Dynamic Light Scattering (DLS) (Zetasizer Nano S, Malvern, UK) was used to investigate the change in hydrodynamic size of the functionalized LNPs. To prepare the sample for DLS, the functionalized liposome solution was diluted with D-PBS at pH 7.4 and equilibrated for 5 minutes to obtain an appropriate count rate. The sample was measured at 25 ˚C with 13 repeats in 10 mm diameter cells, at an angle of 137°.

**Zeta Potential**

The zeta potential of the functionalized LNPs was measured using PALS Zeta Potential Analyzer (Brookhaven Instruments Corp., UK) to determine the stability of the samples. To prepare the sample, the functionalized liposome solution was diluted with D-PBS at pH 7.4 and equilibrated for 5 minutes to obtain an appropriate count rate. The sample was measured at 20 ˚C with 6 repeats (20 cycles per run) at a fixed scattering angle of 90 at 659 nm.

### Leakage of payload and pH-dependent release studies

To measure leakage pH-dependent release from the functionalized LNPs, release profiles of the drug were obtained by placing the samples into dialysis membranes (Float-A-Lyzer®, MWCO 300 kDa, Spectrumlabs, USA). The dialysis membranes were placed in 100 mL PBS buffer at pH 7.4 to measure leakage, and pH 6.5 to measure pH-dependant release. 3 mL samples were taken from the bulk buffer solution at set time intervals and 3 mL of PBS was replaced to maintain the concentration gradient. The fluorescence of the samples containing Calcein was measured using a Spectrofluorometer (GloMax®-Multi Detection System, Promega, USA) at excitation wavelength 495 nm and emission wavelength 515 nm.

### Haemolysis Assay

The endosomolytic behavior of the functionalized LNPs was determined by measuring the membrane disruptive behavior using a hemolysis assay. Samples to be tested were prepared at pH 7.4 and pH 6.5 in D-PBS. Defibrinated sheep red blood cells (RBCs) were washed thrice with D-PBS to obtain a pellet of RBCs. The RBCs were mixed into the samples ensuring a concentration of approximately $2 \times 10^8$ RBCs $mL^{-1}$ was maintained across all samples. This was determined by a calibration line. The samples were incubated for 1 hour at 37 ˚C in a water bath with gentle

agitation. The samples were then centrifuged at 3000 rpm for 3 minutes and the UV absorbance of the supernatants were measured using a UV-Vis spectrophotometer (GENESYS™ 10S UV-Vis spectrophotometer, Thermo Scientific, USA) at 541 nm. The measured absorbances were used to calculate relative hemolysis.

Loading of LNPs into Nanohole Array

**Dry system**

Samples were initially treated with UV ozone (Ossila UV ozone machine) for 15 mins to increase the hydrophilicity of the surface and clean the samples before spin coating. 100 µL of Calcein-loaded LNPs (concentration $1 \times 10^{4.5}$ particles $mL^{-1}$) were first drop cast onto $1 \times 1$ $cm^2$ substrates. Samples were first spun at 500 rpm for 30 s (Ossila spin coater) to spread the drop cast solution before ramping the speed to 3000 rpm (ramp speed, 100 rpm $s^{-1}$) for 60 s until substrate is dry.

**Wet system**

Samples were drop cast with liposomal solution ($1 \times 10^{4.5}$ particles $mL^{-1}$) and allowed to settle for 1 hour in a humid environment to prevent evaporation. This was followed by a cleaning step consisting of a lateral insertion of 100 µL of pure DPBS solution into the drop meniscus before removing excess liquid from the opposite side of the substrate, this procedure was repeated 10 times to remove all excess LNPs from the sample surface. Samples were then sandwiched with a glass cover slip before imaging.

QCM Loading

The silica dioxide (QSX 303, Q-sense) and aluminium oxide (QSX 309, Q-sense) sensors were purchased from Biolin Scientific. Before testing, sensors were flowed over in the sensing module with a peristaltic pump, flowing deionized (DI) water and afterwards DPBS, brought in at a flow rate of 98 μL $min^{-1}$. pH was adjusted by adding 0.1M HCl and NaCl (Sigma Aldrich, Gillingham, United Kingdom), respectively. Before testing, the crystals were cleaned in an iSonic ultrasonic bath with ethanol for 20 minutes and then rinsed with DI water. They were then dried via flowing nitrogen gas. A flow module (QFM 401, Q-sense) was used for testing, whilst keeping the temperature controlled (25 ˚C), flow rate was controlled with a peristaltic pump (Ismatec, ISM795).

The basic model for QCM loaded mass calculations is based upon the Sauerbrey Equation, see Eq.1. When a layer with mass relatively smaller than the quartz crystal binds firmly and evenly on the sensor surface, which is thin and rigid, this model can be used.[4] For a laterally homogeneous film, the Sauerbrey Equation holds when the ratio of $|\Delta D_n/(\Delta f_n/n)|$ is lower than $4 \times 10^{-6}$ $Hz^{-1}$ for a 5 MHz crystal. Otherwise, the viscoelastic models can be applied:[5-7]

$$\Delta m = -\, C \cdot \frac{\Delta f}{n} \tag{1}$$

where Δf is the change of resonance frequency and Δm is the change of loaded mass. C is the mass sensitivity constant (17.7 ng $cm^{-2}$ Hz−1 at the oscillation frequency of 5 MHz), only determined by the intrinsic properties of quartz. n is the odd harmonic number (1, 3, 5, . . .).

Optical Characterization

Scanning electron microscopy was carried out using a Gemini 1 Zeiss Sigma 300. LNP loaded nanohole arrays were pre-sputtered with 10 nm gold with a Quantum Q150T benchtop sputterer.

Confocal microscopy was carried out using a Zeiss LSM-510 inverted laser scanning confocal microscope, 63 × oil objective, at excitation wavelength 488 nm and emission wavelength 535 nm.

## Additional Data

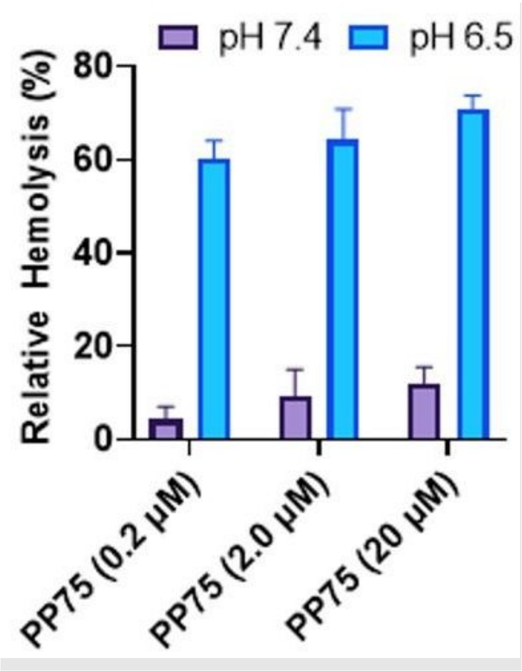


**Figure S1.** Relative hemolysis of RBCs incubated with a negative control of PP75 at various concentrations for 1 h, at pH 7.4 and pH 6.5.

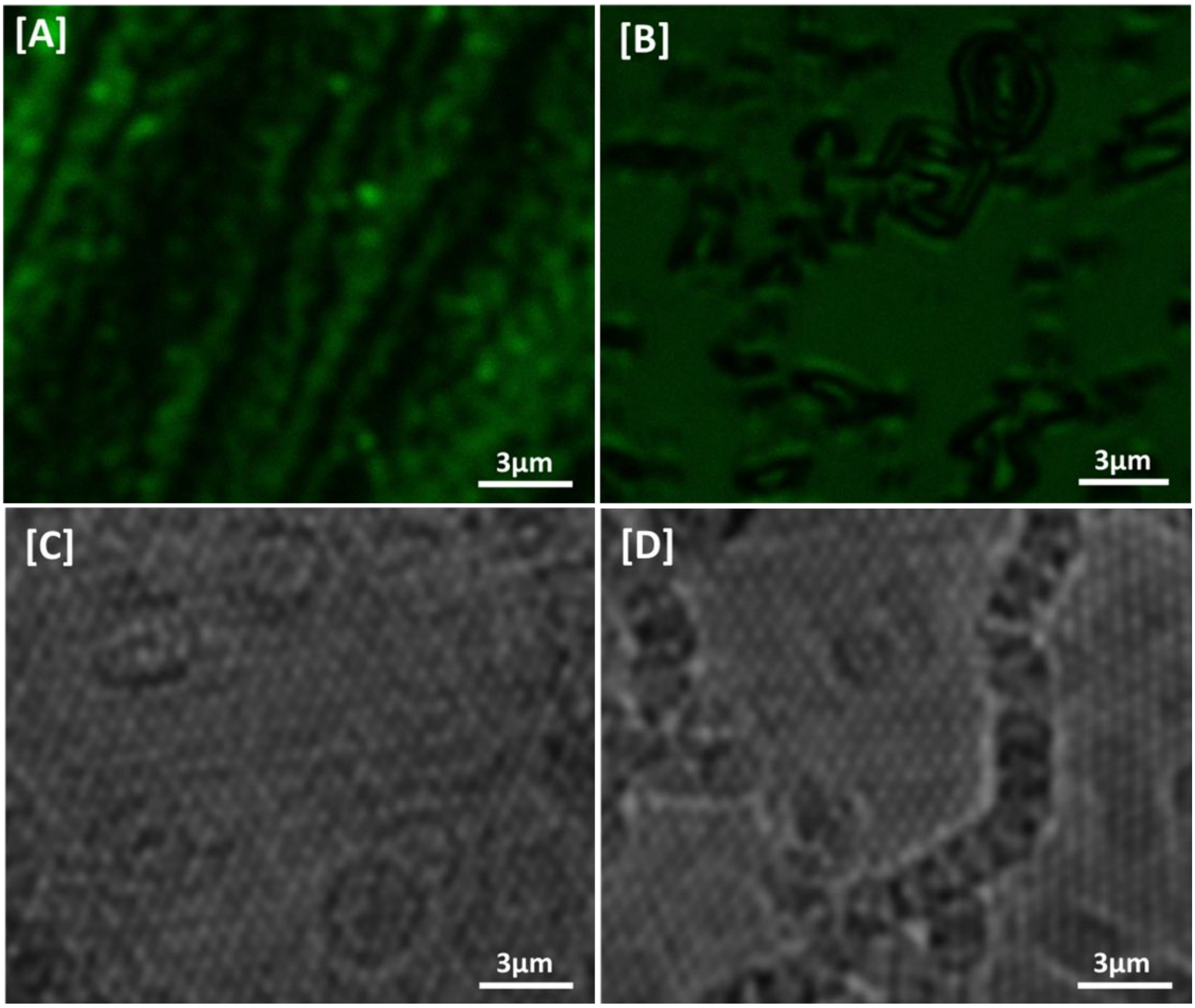


**Figure S2.** [A] Confocal image of flat aluminium film after LNP loading by spin coating, scale bar 3 μm. [B] Confocal image of nanohole array after LNP loading and subsequent washing step, scale bar 3 μm. [C+D] Optical images corresponding to Fig.1A and B, respectively. Scale bars 3 μm.

In Figure S2A the streaked lines shown are due to the precipitation of the salts from DPBS solution after drying.

## References

(1) Chen, R.; Khormaee, S.; Eccleston, M. E.; Slater, N. K. H. The Role of Hydrophobic Amino Acid Grafts in the Enhancement of Membrane-Disruptive Activity of PH-Responsive Pseudo-Peptides. Biomaterials 2009, 30 (10), 1954–1961.

https://doi.org/10.1016/j.biomaterials.2008.12.036.

(2) Chen, R.; Eccleston, M. E.; Yue, Z.; Slater, N. K. H. Synthesis and PH-Responsive Properties of Pseudo-Peptides Containing Hydrophobic Amino Acid Grafts. J. Mater. Chem. 2009, 19 (24), 4217–4224. https://doi.org/10.1039/b902822f.

(3) Guo, F.; Yu, M.; Wang, J.; Tan, F.; Li, N. Smart IR780 Theranostic Nanocarrier for Tumor-Specific Therapy: Hyperthermia-Mediated Bubble-Generating and Folate-Targeted LNPs. ACS Appl. Mater. Interfaces 2015, 7 (37), 20556–20567. https://doi.org/10.1021/acsami.5b06552.

(4) Johannsmann, D. Essentials of Viscoelasticity; Springer, Cham, 2015; pp 33–47. https://doi.org/10.1007/978-3-319-07836-6_3.

(5) Reviakine, I.; Johannsmann, D.; Richter, R. P. Hearing What You Cannot See and Visualizing What You Hear: Interpreting Quartz Crystal Microbalance Data from Solvated Interfaces. Anal. Chem. 2011, 83 (23), 8838–8848. https://doi.org/10.1021/ac201778h.

(6) Easley, A. D.; Ma, T.; Eneh, C. I.; Yun, J.; Thakur, R. M.; Lutkenhaus, J. L. A Practical Guide to Quartz Crystal Microbalance with Dissipation Monitoring of Thin Polymer Films. J. Polym. Sci. 2022, 60 (7), 1090–1107. https://doi.org/10.1002/pol.20210324.

(7) Srimasorn, S.; Souter, L.; Green, D. E.; Djerbal, L.; Goodenough, A.; Duncan, J. A.; Roberts, A. R. E.; Zhang, X.; Débarre, D.; DeAngelis, P. L.; Kwok, J. C. F.; Richter, R. P. A Quartz Crystal Microbalance Method to Quantify the Size of Hyaluronan and Other Glycosaminoglycans on Surfaces. Sci. Rep. 2022, 12 (1), 10980. https://doi.org/10.1038/s41598-022-14948-7.
